# Factors influencing the behaviour and perceptions of Australian veterinarians towards antibiotic use and antimicrobial resistance

**DOI:** 10.1371/journal.pone.0223534

**Published:** 2019-10-10

**Authors:** Jacqueline M. Norris, Annie Zhuo, Merran Govendir, Samantha J. Rowbotham, Maurizio Labbate, Chris Degeling, Gwendolyn L. Gilbert, Dale Dominey-Howes, Michael P. Ward

**Affiliations:** 1 Sydney School of Veterinary Science, The University of Sydney, Sydney, New South Wales, Australia; 2 Westmead Institute for Medical Research, Sydney, New South Wales, Australia; 3 School of Geosciences, The University of Sydney, Sydney, New South Wales, Australia; 4 Menzies Centre for Health Policy, Sydney School of Public Health, The University of Sydney, Sydney, New South Wales, Australia; 5 School of Life Sciences, University of Technology Sydney, Sydney, New South Wales, Australia; 6 Australian Centre for Health Engagement, Evidence & Values, School of Health and Society - Faculty of Social Sciences, University of Wollongong, Wollongong, New South Wales, Australia; 7 Wollongong Antimicrobial Resistance Research Alliance (WARRA), University of Wollongong, Wollongong, New South Wales, Australia; 8 Marie Bashir Institute for Infectious Diseases and Biosecurity, The University of Sydney, Sydney, New South Wales, Australia; The University of Melbourne, AUSTRALIA

## Abstract

Antimicrobial resistance (AMR) is a global crisis with impacts on the future health and welfare of humans and animals. Determining key factors that influence veterinarians’ antimicrobial prescribing behaviours can bridge the gap between prescribing guidelines and clinical usage. Veterinarians practicing in Australia were surveyed on their frequency in prescribing different antibiotics; factors influencing their antibiotic prescribing behaviours; and their perceptions of current drivers of AMR. Antibiotics were prescribed in a third of consultations with key differences in the frequency of use of specific antibiotics by small companion animal (SCA), equine and livestock veterinarians, which broadly aligned with antibiotic registration restrictions in Australia. SCA veterinarians reported prescribing broad-spectrum antibiotics of higher importance to human health more frequently than livestock veterinarians. Factors that were reported as ‘strong’ or ‘moderate’ barriers to appropriate antibiotic prescribing were the 1) cost of culture and susceptibility testing and 2) lack of access to rapid and affordable diagnostic tests. Fear of losing clients, colleague pressure, and lack of their own understanding about antibiotics were considered to be ‘no’ or ‘somewhat’ of a barrier to appropriate prescribing by respondents. SCA veterinarians placed greater importance on the contribution of antibiotic use in livestock to AMR, than antibiotic use in companion animals. Despite reporting use of fewer, mostly narrow spectrum antibiotics of lower importance to human and animal health, livestock veterinarians were generally more aware of their potential contribution to AMR. This study provides insights into the similarities and differences in SCA, equine and livestock veterinarians practicing in Australia and informs sector-specific strategies to improve antimicrobial stewardship.

## Introduction

Antimicrobial resistance (AMR) is a critical global health issue with potentially far-reaching impacts on the health of humans and animals [1]. The global need to reduce antimicrobial use and thereby reduce selective pressures on microbes, is driving research on factors influencing the antimicrobial prescribing behaviours of health professionals [2,3].

Following the recommendations of the Swann report [4], Australia was one of a few countries to restrict the legal prescription of antimicrobials to certain professions: dentists, doctors, veterinarians and later, nurse practitioners. In Australia, national practice-level therapeutic guidelines for antimicrobial use are widely available for doctors, nurse practitioners and dentists [5]. Assessment of antimicrobial prescribing practices in these professions has been actively performed via a range of audits. Australian veterinarians have had limited guidance for antimicrobial prescribing. Examples include evidence-based consensus statements on specific common diseases [6–9], production-industry guidelines as part of quality assurance programs, and exclusion of many antimicrobial agents for use in livestock and horses by the Federal Government Agency—the Australian Pesticides and Veterinary Medicines Authority (APVMA). However, monitoring or auditing the antimicrobial prescribing practices of Australian veterinarians has not been published to date.

Our ability to capture the quantity and context of antimicrobial prescribing by veterinarians in Australia is currently limited to import quantities collated in periodic reports produced by the APVMA [10]. Recent studies demonstrate substantial variation in Australian veterinarians’ compliance with guidelines or accepted standards, depending on species and the patient’s clinical circumstances [11–15], but did not explore concurrently, influences and barriers to their prescribing behaviour or attitudes to AMR. To increase the effectiveness of antimicrobial prescribing, we need to further understand the drivers and barriers to responsible and appropriate administration by veterinarians in different practice types.

Globally, a range of both extrinsic and intrinsic factors—beyond scientific knowledge and clinical evidence—influence veterinarians. These include veterinarians’ antimicrobial preferences and professional experience; perceived antimicrobial efficacy; ease of patient administration; perceived owner compliance; animal characteristics; suboptimal housing conditions; biosecurity measures; cost of diagnostic tests; and social norms within clinical practices in Australia [16], the United Kingdom [17], the USA [18], Ireland [19], New Zealand [20], Belgium [21], the Netherlands [22] and Europe more broadly [23,24]. Research that relates the frequency of antimicrobial usage within different veterinary practice types is critical to the success of international and national efforts to manage antimicrobial resistance [25].

Factors influencing veterinarian prescribing behaviour and their perceptions of who contributes to the AMR issue needs to be better understood. We recently reported that prescribers of antibiotics tend to externalise the concept of who is most responsible for AMR, placing the contribution of other individuals (ie., other veterinarians) and professional sectors (ie., doctors and dentists) before their own [3]. Within the veterinary sector, there might be great variation in ‘attribution of blame’ within the profession due to the diverse nature of species treated and a general stratification of the workforce. The animal’s societal role is also likely important: companion animal (a.k.a. pet) or economic commodity and component of the food and fiber chain. To ensure the success of national and international efforts to reduce AMR, it is essential to concurrently compare the frequency of antimicrobial usage amongst different veterinary practice types and directly relate this to factors influencing their prescribing behavior—something not yet addressed in Australia but formally recognized as lacking [25]. Given the diverse nature of the veterinary profession, it is necessary to further assess the differences in how members of each veterinary practice type perceive the role of their own and other practice types in contributing to AMR [16].

Considering these priorities, our aim was to compare small companion animal (SCA), equine and livestock veterinarians with respect to their frequency in prescribing a range of different antibiotics; factors influencing their antibiotic prescribing behaviours; and their perceptions of current drivers of antimicrobial resistance.

## Methods

### Recruitment

Self-administered surveys were hosted on SurveyMonkey^™^ and distributed online between October and December 2016. Veterinary respondents were contacted *via* professional associations (the Australian Veterinary Association, the Australian and New Zealand College of Veterinary Scientists), state practitioner boards (required for veterinary registration) and other professional organisations using newsletters, bulletins, professional e-mail lists, forum announcements, social media sites and websites.

### Study design

A cross-sectional study was designed to target veterinarians registered in Australia across all sectors of the profession as part of a broader survey of antibiotic prescribing professionals in Australia (doctors, dentists, veterinarians) [3]. Survey questions were developed based on review of the relevant literature; designed in collaboration with experienced doctors, dentists and veterinarians; and pre-tested and refined with feedback from practitioners. Feedback on the questionnaire design was received from the Office of Health Protection, The Department of Health, Australian Federal Government, who co-lead Australia’s AMR Strategy 2015–2019 and its implementation plan, to ensure data generated were relevant and useful for national decision-makers.

The survey comprised 45 questions: three open questions and 42 closed or semi-closed questions, mostly with Likert-type responses measured on four or five-point scales. “Unsure”, “neutral” and “not applicable (N/A)” options were also provided for specific questions, where appropriate. The survey was divided into four sections: information about the respondent and their practice; antibiotic prescribing behaviour and influences; information sources; and perceptions regarding antibiotics and antibiotic resistance. A copy of the survey is available as an online supplement (S1 Survey) and was previously published as an online supplement [3]. The University of Sydney Human Research Ethics Committee (Project Number: 2016/675) approved this study.

Demographic data was collected at two levels: a) *veterinarian-level characteristics* including age, gender, years of veterinary practice experience (including year of graduation), place of graduation, and level of postgraduate education and whether the respondent had specialisation credentials; and b) *practice-level characteristics* including the type of veterinary practice (percentage of each animal species treated; animal species most frequently treated), and practice location (postcode). To determine their antibiotic prescribing behaviour, respondents were asked to nominate the animal species they treated most frequently and to estimate the frequency with which they administered or dispensed a range of different antibiotics to this species from ‘never’ to ‘frequently’ (1 = Never, 2 = Rarely, 3 = Sometimes, 4 = Frequently). Responses to which animal species they most frequently treated were coded according to practice type as *small companion animal* (SCA; dogs, cats, pocket pets), *equine* (horses) and *livestock* (cattle, sheep, goats, pigs, poultry). Livestock were grouped together due to the absence of APMVA registration for many antibiotics of high importance in these species.

**Fig 1:**
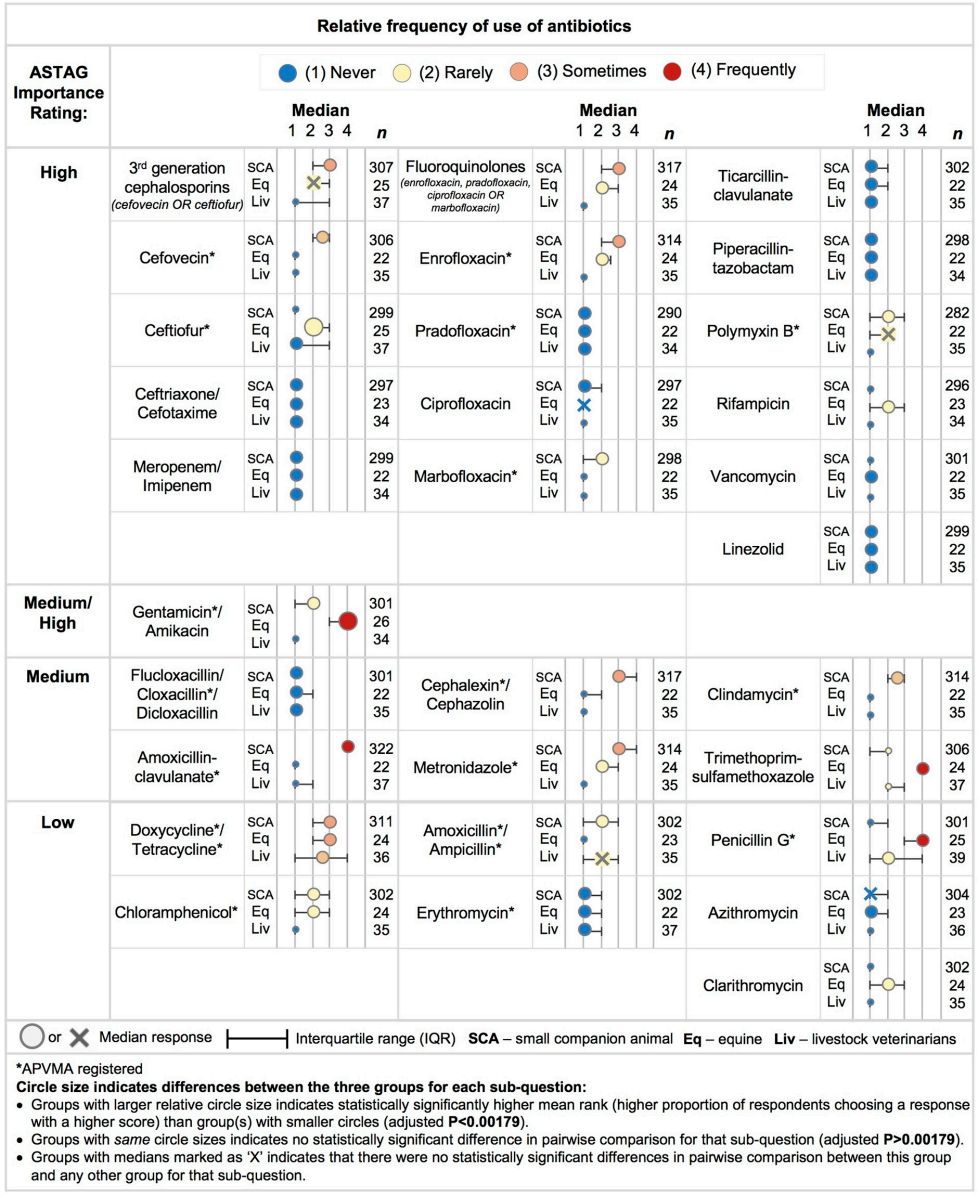
Frequency of antibiotic use relative to type of practice (small companion animal [SCA], equine [Eq], livestock [Liv]) with the antibiotic classified by the Australian strategic and technical advisory group (ASTAG) rating of importance to human health [26].

**Fig 2:**
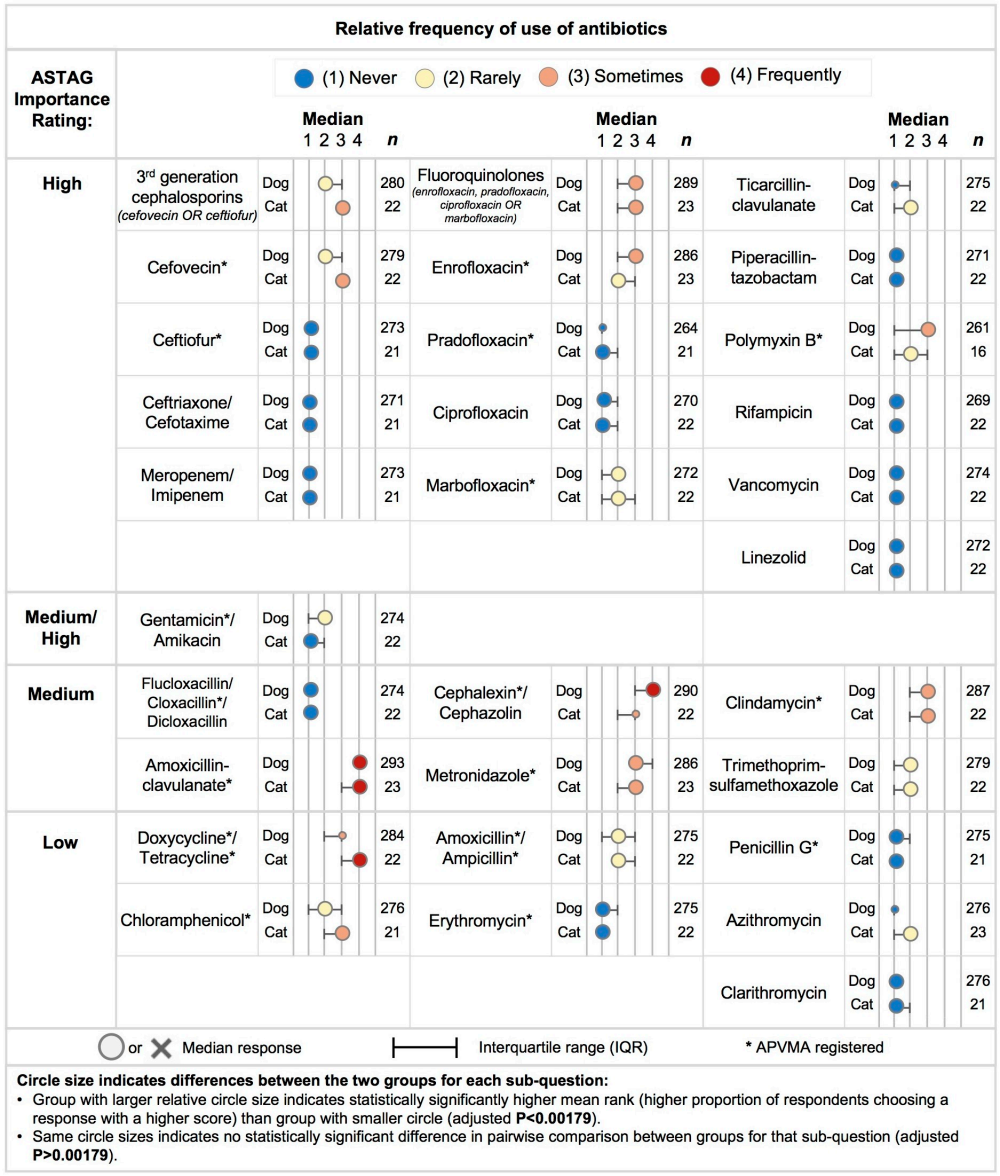
Frequency of antibiotic use relative to type of practice (veterinarians who predominantly treat dogs compared to those who predominantly treat cats) with the antibiotic classified by the Australian strategic and technical advisory group (ASTAG) rating of importance to human health [26].

For analysis of other questions (other than the question about relative frequency of prescribing/administering a range of different antibiotics; Figs 1 and 2), respondents were classified according to practice type as ‘*small companion animal*’ (SCA; dogs, cats, pocket pets), ‘*equine*’ (horse) and ‘*livestock*’ (cattle, sheep, goats, pigs, poultry) if the responding veterinarian spent more than 50% of their time treating animal species in that group. Those who did not indicate that they spent at least 50% of their time with any particular species were categorised according to the most common animal species they treated. Antibiotics were listed by their non-proprietary name, and if applicable, followed by a range of common veterinary drugs in that class, available in Australia. During analysis, the antibiotics were ranked as ‘high’, ‘medium’ or ‘low’ in accordance with the Australian Strategic and Technical Advisory Group (ASTAG) on importance rating for the mitigation of antibacterial resistance [26] and this classification is used in Fig 1. The focus of the study was antibiotic resistance (AbR) rather than AMR, due to the common use of antibiotics to treat animals and humans. The term antibiotic is used as it is in common usage to refer to medicines used to prevent and treat bacterial infections [27].

### Statistical analyses

Statistical analyses were conducted using IBM SPSS Statistics version 22. Respondents completing the demographic questions and at least one other question were included in the analyses. If a response was missing for a single item, the item was excluded from that respective analysis (list-wise deletion). Medians and interquartile ranges (IQRs) were calculated and reported for questions with an ordinal response scale. Kruskal-Wallis H statistical tests were conducted to determine significant differences in median responses and mean ranks between different veterinarian groups; *post hoc* analyses of pairwise comparisons were performed using Dunn’s (1964) procedure, with a Bonferroni correction for multiple comparisons to estimate adjusted p-values. Kruskal-Wallis H statistical tests were also used to compare demographic sub-groups (e.g. specialists vs. non-specialists) within the group of veterinarians who mainly treated dogs (as this was the largest respondent group) as well as the relative frequency of this group’s prescription of different antibiotics for their patients. All “N/A” and “Unsure” responses were excluded from all statistical analyses. To compare mean antibiotic prescribing frequencies between the practice types, ANOVA was used. For all tests statistical significance was p < 0.05. The results of all analyses were collated (S1 Table).

## Results

### Respondent characteristics

Overall, responses were received from 403 veterinarians, representing 3.9% of the Australian national veterinary workforce. Of these responses, three veterinarians did not indicate which species they treated in their current or most recent role. Additionally, 13 respondents indicated they predominantly treated species (such as wildlife and zoo animals) other than small companion animals (SCA), equine or livestock. Consequently, 16 responses were excluded from the statistical analysis. The remaining 387 responses were from veterinarians who spend most of their time treating SCA (320, 82.7%), horses (26, 6.7%) or livestock (41, 10.6%) (Table 1). This is broadly representative of the national veterinary workforce [28].

**Table 1:** General characteristics of survey respondents by veterinarian practice type in a national survey of antibiotic use by Australian veterinarians, 2016.

	Small companion animal (SCA)		Equine (Eq)		Livestock (Liv)		TOTAL	
	n	%	n	%	n	%	n	%
**SAMPLE**	320	82.7	26	6.7	41	10.6	387	100.0
**State/territory (of principal place of practice (PPP))**								
New South Wales (NSW)	85	26.6	9	34.6	12	30.0	106	27.5
Victoria (VIC)	66	20.7	2	7.7	13	32.5	81	21.0
Queensland (QLD)	25	7.8	5	19.2	3	7.5	33	8.6
South Australia (SA)	6	1.9	1	3.8	2	5.0	9	2.3
Western Australia (WA)	79	24.8	7	26.9	5	12.5	91	23.6
Tasmania (TAS)	31	9.7	0	0.0	0	0.0	31	8.1
Northern Territory (NT)	2	0.6	0	0.0	2	5.0	4	1.0
Australian Capital Territory (ACT)	25	7.8	2	7.7	3	7.5	30	7.8
Total	319	100.0	26	100.0	40	100.0	385	100.0
Missing	1		0		1		2	
**Urban/Rural (postcode of PPP)**								
Urban	201	68.1	12	48.0	13	36.1	226	63.5
Rural	94	31.9	13	52.0	23	63.9	130	36.5
Total	295	100.0	25	100.0	36	100.0	356	100.0
Missing	25		1		5		31	
**Gender**								
Female	222	69.6	12	46.2	19	46.3	253	65.5
Male	97	30.4	14	53.8	22	53.7	133	34.5
Total	319	100.0	26	100.0	41	100.0	386	100.0
Missing	1		0		0		1	
**Age**								
<35	113	35.5	9	37.5	15	36.6	137	35.8
35–54	148	46.5	7	29.2	16	39.0	171	44.6
≥55	57	17.9	8	33.3	10	24.4	75	19.6
Total	318	100.0	24	100.0	41	100.0	383	100.0
Missing	2		2		0		4	
**Years of experience as a vet**								
10 years or less	126	39.4	10	38.5	13	31.7	149	38.5
11–20 years	86	26.9	4	15.4	10	24.4	100	25.8
21–30 years	54	16.9	4	15.4	6	14.6	64	16.5
More than 30 years	54	16.9	8	30.8	12	29.3	74	19.1
Total	320	100.0	26	100.0	41	100.0	387	100.0
Missing	0		0		0		0	
**Level of Specialization**								
Non-specialist vet	298	93.1	19	73.1	32	78.0	349	90.2
Specialist vet	22	6.9	7	26.9	9	22.0	38	9.8
Total	320	100.0	26	100.0	41	100.0	387	100.0
Missing	0		0		0		0	
**Main work setting**								
Private practice	273	85.3	18	69.2	18	43.9	309	79.8
University teaching hospital	15	4.7	5	19.2	1	2.4	21	5.4
Other Teaching/Research	12	3.8	1	3.8	4	9.8	17	4.4
Not-for-profit veterinary practice	10	3.1	0	0.0	0	0.0	10	2.6
Government	7	2.2	2	7.7	14	34.1	23	5.9
Industry	3	0.9	0	0.0	4	9.8	7	1.8
Total	320	100.0	26	100.0	41	100.0	387	100.0
Missing	0		0		0		0	

Those responding to the survey represented a range of ages and years of experience as practicing veterinarians, relative to the national workforce [3,28]. More than two-thirds of SCA veterinarians (68.1%) practiced in urban areas, compared to 48.0% of equine and 36.1% of livestock veterinarians. More than two-thirds of SCA veterinarians were female (69.6%), compared to 46.2% of equine and 46.3% of livestock veterinarians. A higher proportion of equine (26.9%) and livestock (22.0%) veterinarians were specialists compared to SCA veterinarians (6.9%). The majority of SCA veterinarians (85.3%) and equine veterinarians (69.2%), and less than half of the livestock veterinarians (43.9%), worked in private practice.

### Frequency of antibiotic prescribing relative to practice type

Respondents reported a median case load of 40 patients per week (IQR: 50 patients). The reported median percentage of antibiotics prescribed / administered to patients per week was 33.3% (mean = 36.1%) with no significant difference found between practice types (p = 0.057). The predominant animal species that veterinary respondents treated in their clinical practice were dogs (n = 293), followed by horses (n = 26), cats (n = 23), cattle (n = 19), goats / sheep (n = 14), pigs (n = 5) and poultry (n = 2).

Among SCA veterinarians, the antibiotics reported as most commonly prescribed / administered were amoxicillin-clavulanate (median–‘frequently’) followed by first generation cephalosporins (cephalexin / cephazolin), metronidazole, doxycycline / tetracycline, enrofloxacin and third generation cephalosporins (median–‘sometimes’) (Fig 1). Considering the antibiotics of high importance to human and animal health [26], the veterinary fluoroquinolone enrofloxacin, and third generation cephalosporin, cefovecin, were reported as being prescribed ‘sometimes’ by SCA veterinarians. Other drugs were ‘rarely’ or ‘never’ used.

For the treatment of dogs, antibiotics that respondents used ‘frequently’ were amoxicillin-clavulanate and cephalexin / cephazolin (Fig 2). Those reportedly used ‘sometimes’ were enrofloxacin, polymyxin B, metronidazole, clindamycin and doxycycline / tetracycline. Antibiotics reported as ‘rarely’ prescribed for dogs included trimethoprim-sulfamethoxazole, amoxicillin / ampicillin, gentamicin / amikacin, chloramphenicol, the third-generation cephalosporin cefovecin and marbofloxacin. The median frequency for all other antibiotics listed were reported as ‘never’. Among those veterinarians who predominantly treated dogs, non-specialists reported more frequent antibiotic prescribing of cefovecin (median–‘sometimes’) than canine specialists (median–‘never’) (p < 0.001). For other important antibiotics used in dogs—including marbofloxacin (p = 0.002), meropenem (p < 0.001) and rifampicin (p < 0.001)—use was more frequent among specialists (median response was ‘rarely’) than non-specialists (median response was ‘never’).

‘Frequently’ prescribed antibiotics specifically for cats were amoxicillin-clavulanate and doxycycline (Fig 2). First generation cephalosporins (cephalexin / cephazolin), metronidazole, clindamycin, the third-generation cephalosporin cefovecin, and chloramphenicol were reported as prescribed ‘sometimes’. Antibiotics reported to be used rarely in cats were the fluoroquinolones, polymyxin B, trimethoprim-sulfamethoxazole, amoxicillin / ampicillin, ticarcillin-clavulanate and azithromycin. The median frequencies for all other antibiotics were reported as ‘never’ prescribed.

Equine veterinarians reported the most ‘frequently’ prescribed / administered antibiotics were trimethoprim-sulfamethoxazole, penicillin G and gentamicin; followed by tetracycline/doxycycline (median–‘sometimes’) (Fig 1). Antibiotics that were reported as ‘rarely’ prescribed, were metronidazole, ceftiofur, rifampicin, chloramphenicol, enrofloxacin, polymixin B and clarithromycin. The median frequencies for all other antibiotics listed in the question were reported as ‘never’.

Among livestock veterinarians, the frequency of antibiotic use was highly variable. Antibiotics reported as most commonly prescribed / administered were tetracycline / doxycycline (median–‘rarely to sometimes’; IQR ‘never’ to ‘frequently’), penicillin G (median–‘rarely’; IQR ‘‘never’ to ‘frequently’), amoxicillin / ampicillin (median–‘rarely’; IQR ‘never’ to ‘sometimes’) and trimethoprim-sulfamethoxazole (median–‘rarely’; IQR ‘rarely’ to ‘sometimes’). The highest reported use of ceftiofur was ‘frequently’ (median–‘never’; IQR–‘never to sometimes’). In general, for antibiotics of high importance to human and animal health, the median frequency of reported use was ‘never’, whereas for antibiotics of ‘medium’ or ‘low’ human health importance, the median frequency of use in livestock was up to ‘rarely’ or ‘sometimes’, respectively.

### Factors influencing veterinarians’ antibiotic prescribing behaviour

**Fig 3:**
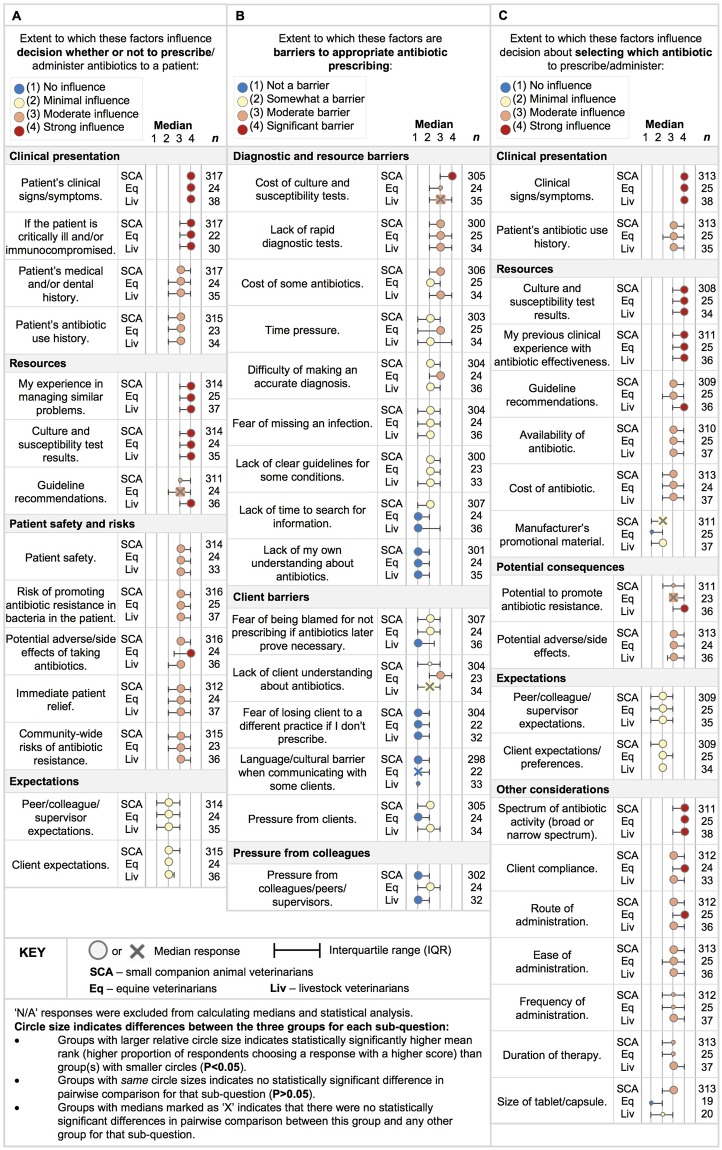
Factors influencing antibiotic prescribing among small companion animal [SCA], equine [Eq], and livestock [Liv] veterinarians.

Factors reported as ‘strongly’ or ‘moderately’ influencing antibiotic prescribing for all practice types were the patient’s clinical signs and medical history; the critical nature of patient illness; the patient’s antibiotic use history; the veterinarian’s experience in managing similar problems; culture and susceptibility testing results; patient safety; risk of promoting AMR in the patient or in the community; antibiotic potential for inducing adverse effects and to provide immediate patient pain relief (Fig 3A–3C). Factors rated as having ‘minimal’ influence for most veterinarians were clients’ or colleagues’ expectations. Livestock veterinarians generally indicated guideline recommendations as having a ‘strong’ influence on their decisions, while SCA and equine veterinarians rated guideline recommendations as a ‘moderate’ influence.

Factors that were a ‘significant’ or ‘moderate’ barrier to appropriate antibiotic prescribing for all practice types were the cost of culture and susceptibility testing and lack of rapid diagnostic tests (Fig 3B). Factors rated as either a ‘moderate’ or ‘somewhat’ barrier among practice types included the cost of some antibiotics, time pressure during a consultation / making a diagnosis, difficulty of making an accurate diagnosis, fear of missing an infection, and lack of clear guidelines for treating some conditions. Factors generally considered to be ‘no’ or ‘somewhat’ a barrier to appropriate prescribing were fear of losing clients to another practice, pressure from colleagues, lack of respondents’ own understanding about antibiotics, fear of being blamed for not prescribing antibiotics and language/cultural barriers when communicating with clients. SCA veterinarians were more likely to regard the ‘cost of culture and susceptibility tests’ as a ‘significant’ barrier. Equine veterinarians rated ‘lack of client understanding of antibiotics’ as a ‘moderate’ barrier, while SCA and livestock veterinarians rated this ‘somewhat’ of a barrier. Although the majority of respondents (88.1%) indicated that they have felt pressure / expectations from their clients to prescribe / administer antibiotics to their animals (SCA—88.7%; Equine—92%, Livestock—81.1%); SCA and livestock veterinarians considered it ‘somewhat’ of a barrier and equine veterinarians considered it ‘not’ to be a barrier to appropriate prescribing, and all practice types reported their client’s expectations had ‘minimal’ influence on whether or not to prescribe.

Approximately half of all SCA (49.3%; 150/304), equine (56.5%; 13/23), and livestock veterinarians (51.4%; 18/35) indicated that there were some antibiotics they do not feel comfortable prescribing or administering. These included fluoroquinolones (n = 88), third generation cephalosporins (n = 46), vancomycin (n = 19), chloramphenicol (n = 12) and carbapenems (n = 21). Reasons included the importance of these drugs in human medicine or recognition of these as last-line antibiotics (n = 24); need to ensure their use is supported by culture and susceptibility testing (n = 9) and concerns regarding promotion of antimicrobial resistance (n = 7).

An additional factor generally rated as ‘strongly’ influencing selection of specific antibiotics by all practice types was the spectrum of antibiotic activity (broad vs narrow spectrum) (Fig 3C). An additional factor rated as having ‘no’ or ‘minimal’ influence was manufacturer promotional material. Livestock veterinarians were more likely than SCA (p<0.05) and equine veterinarians (p<0.05) to indicate that ‘frequency of administration’ and ‘duration of therapy’ had a ‘moderate’ influence on their selection of antibiotic. Livestock veterinarians were more likely than SCA veterinarians to rate ‘potential to promote antibiotic resistance’ as having a ‘strong’ influence on their decision (p = 0.025) on specific antibiotic choice. Equine veterinarians considered ‘client compliance’ and the ‘route of administration’ had a ‘strong’ influence on their specific antibiotic agent prescribing.

### Perceptions of current drivers to antimicrobial resistance

**Fig 4:**
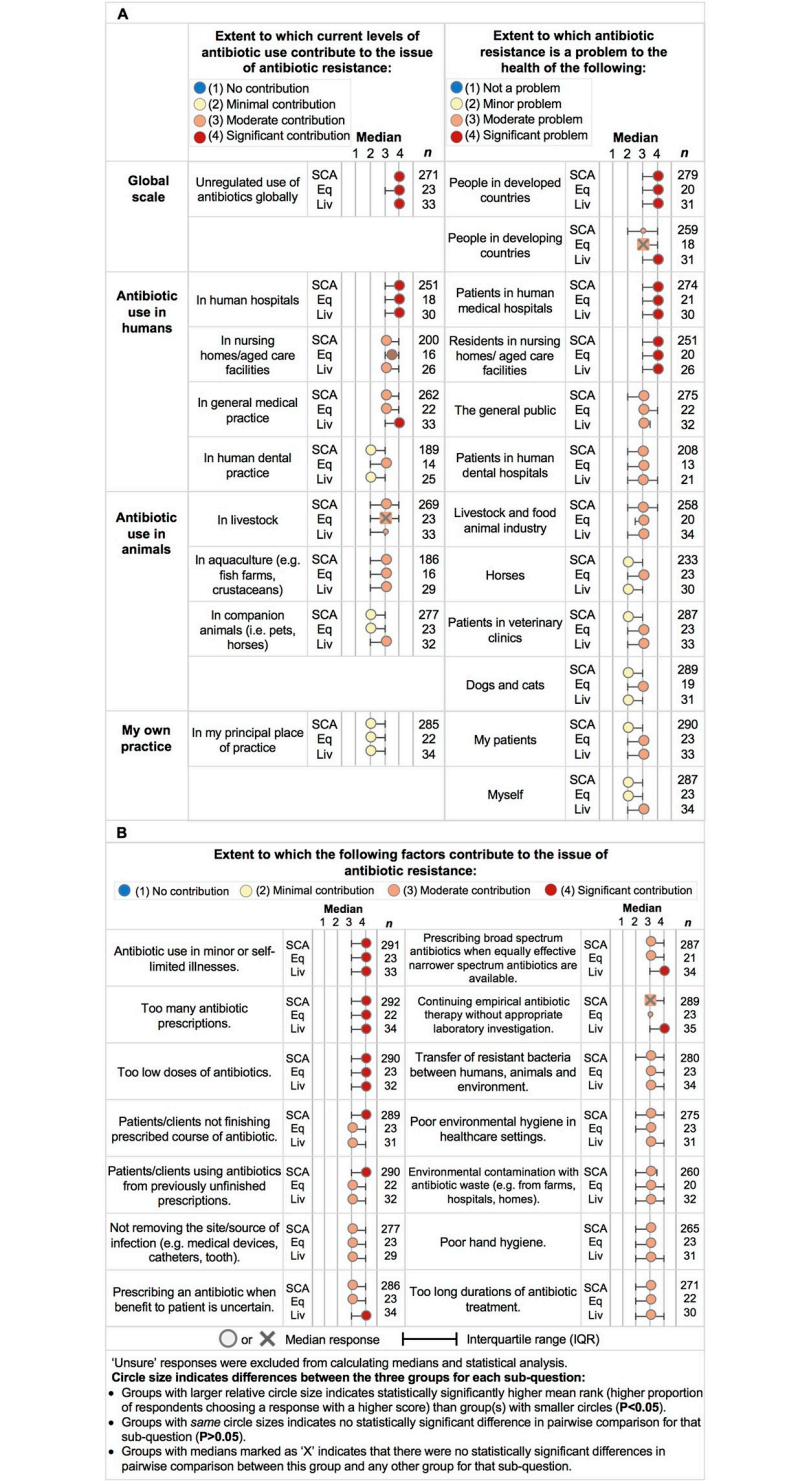
Perceptions among small companion animal [SCA], equine [Eq], and livestock [Liv] veterinarians about the contribution of various factors to antibiotic resistance and the extent to which it is a problem to the health of various populations.

The majority of veterinarians perceived current levels of antibiotic use in human hospitals, nursing homes / aged-care facilities, general medical practice and unregulated use of antibiotics globally to be a ‘significant’ or ‘moderate’ contributor to the issue of antibiotic resistance, and a ‘significant’ problem for people in developed countries (Fig 4A). Livestock veterinarians were more likely than SCA veterinarians to perceive antibiotic resistance as a ‘significant’ problem to the health of people in developing countries (p = 0.010). All practice types generally considered antibiotic use in aquaculture to be a ‘moderate’ contributor to AMR. Compared to SCA veterinarians, a lower proportion of livestock veterinarians perceived current levels of antibiotic use in livestock as a ‘significant’ contributor to the issue of antibiotic resistance (p = 0.041). SCA and equine veterinarians considered antibiotic use in companion animals (dogs, cats, horses) to have ‘minimal’ contribution to AMR, whereas livestock veterinarians considered it ‘moderate’. All practice types considered their own practice as contributing minimally to the issue of AMR. Overall, the three veterinary practice types considered AMR a ‘moderate’ problem for the general public.

Perceptions of the threat of antibiotic resistance to veterinary patients were more mixed between practice types but generally perceived as a problem. All practice type groups agreed that AMR was a ‘moderate’ problem in livestock and the food industry. Equine veterinarians considered AMR in horses, dogs and cats to be a ‘moderate’ problem whereas SCA and livestock veterinarians considered it to be a ‘minor’ problem. The majority of SCA (80.9%), equine (73.9%) and livestock (69.4%) veterinarians have seen antibiotic-resistant infections in patients and have experienced treatment failure with antibiotics. However, SCA veterinarians, and both equine and livestock veterinarians considered AMR to be a ‘minor’ and ‘moderate’ problem in their patients, respectively.

All practice types agreed that ‘antibiotic use in minor or self-limited illnesses’, ‘too many prescriptions’ and ‘too low doses of antibiotics’ contributed ‘significantly’ to antimicrobial resistance. Veterinarians generally thought that patients / clients ‘not finishing prescribed course of antibiotics’, ‘not removing the site/source of infection’, prescribing an antibiotic when the benefit to the patient is uncertain’ and ‘using antibiotics from a previously unfinished course’ was a ‘moderate’ or ‘significant’ contributor to antibiotic resistance. All practice types generally agreed that ‘prescribing broad spectrum antibiotics when equally effective narrower spectrum antibiotics are available’; ‘too long duration of antibiotic treatment’ and ‘continuing antibiotics without appropriate laboratory investigation’ were a ‘significant’ to ‘moderate’ contributor to AMR. There was general agreement that environmental sources through environmental contamination with antibiotic waste, poor hand and healthcare hygiene, and transfer of resistant bacteria between humans, animals and the environment to be ‘moderate’ contributors to AMR.

## Discussion

Key differences in the reported frequency of prescribing specific antibiotics by the three veterinarian groups were discovered in this study. There was commonality in factors influencing antibiotic prescribing behaviour; but there were some important disparities in the perception of respondents regarding current drivers of AMR. This information can be used when developing prescriber guidelines and sector-specific stewardship programs for Australian veterinarians.

Current antimicrobial prescribing guidelines in veterinary practice focus mainly on clinical, microbiological and pharmacological indications for prescribing [29]. The current study highlights the importance of patient-centric (patient’s clinical signs and history, critical nature of their illness, patient safety, patient’s antibiotic use history, potential for adverse effects), microbial-centric (results of culture and susceptibility testing), and veterinarian-centric factors (their experience in treating similar clinical signs) influencing whether or not the three groups of Australian veterinarians prescribe antibiotics. Some of these factors have been previously identified as key drivers for deciding whether to prescribe antibiotics [23].

Survey respondents reported their clients’ or colleagues’ expectations had ‘no’ or ‘minimal’ influence despite a high proportion perceiving client pressure to prescribe. Using semi-structured interviews [16], Hardefeldt and colleagues found that veterinarians felt pressured by clients to offer some form of treatment and a subset of clients expected antibiotics without a formal consultation. They reported that some veterinarians felt pressure to satisfy clients, because of competition between practices, especially among equine and cattle practices. Smith and colleagues [30] in semi-structured interviews of pet owners and veterinarians in the UK interestingly found a disconnect between the perceptions of others positions and intentions with vets perceiving that pet owners pushed for antibiotics while pet owners felt they were overprescribed. It is essential that preclinical and clinical veterinary training incorporates the principles of antimicrobial stewardship to ensure veterinarians continue to discuss with clients the need to preserve antimicrobial efficacy [31]. Broader community-based awareness programs through initiatives in the human health care sector may also have an impact of veterinary client expectations as the community becomes increasingly aware of the need to use antibiotics prudently.

The cost of culture and antimicrobial susceptibility testing (AST); lack of rapid, affordable diagnostic tests for determining whether antibiotics are required; and identifying the most suitable antibiotic for patients’ infections, have been identified globally as a consistent barrier to the appropriate prescribing of antibiotics by veterinarians [23,32]. While veterinarians in all practice types remain concerned about the risk of promoting AMR in their patients or within the community, cost remains a barrier to obtaining microbial culture and AST to guide antimicrobial choice [18,32]. Studies of the decision-making processes of European veterinarians when choosing antibiotics to prescribe [23] found AST was usually performed following empirical treatment failure. With the privatisation of most veterinary diagnostic laboratories in Australia, the geographic centralisation of AST by some diagnostic laboratory groups and the large distances in Australia, amelioration of the cost of AST in veterinary practice in Australia and the development of better in-clinic testing through technological advances, will require both financial and professional subsidisation and support. The results of our study reinforce the argument that prescribing guidelines that include diseases and procedures in which antibiotics should not be used and evidence-based guidelines on duration of therapy when antimicrobial agents are required, are essential [9].

The frequency of prescribing specific antibiotics reported by veterinarians in this survey broadly reflected the availability and restrictions imposed in Australia by the APVMA on the registration of antibiotics for use in animals. The only antibiotic of critical importance registered for use in livestock in Australia is the third-generation cephalosporin ceftiofur, registered for use in cattle for bovine lower respiratory disease and single use for foot abscesses. Conversely a range of high importance antibiotics—such as the fluoroquinolone class (enrofloxacin, marbofloxacin, pradofloxacin)—are registered for use in dogs and cats, while the third-generation cephalosporin ceftiofur is registered for use in dogs and horses, and cefovecin is registered for some soft tissue and urinary infections in dogs and cats in Australia. Under the legislation in the Stock Medicines Act governed by each state in Australia, veterinarians are permitted to use their professional judgement to prescribe antimicrobials off-label only for individual animals and not for conditions affecting animal herds or groups. While the concept of off-label use in veterinary practice has been criticised, current legislation restricts the updating of antimicrobial labelling to reflect current effective doses for common veterinary pathogens [33]. Restriction to on-label dosing would potentially lead to under dosing [33] and highlights the need for legislative changes to ensure antimicrobial drug labels are frequently updated as a key element of improving antimicrobial stewardship in Australia. Our study supports the need for careful sector specific approaches to the development of such initiatives.

The use of antibiotics in production animals, especially food-producing, has been the focus of an increasing number of studies [34–36] and media reports. Global reports on AMR and issues around antimicrobial stewardship in livestock have been commonly extrapolated to Australia without geographic context and qualification of the restricted registration of antimicrobial agents for use in Australian livestock. This current survey found that Australian livestock veterinarians reported the use of a small number of narrow spectrum antibiotics mostly of low importance to human and animal health; drugs of high importance were reported to be rarely used. Livestock veterinarians in this survey were more cognisant of their potential contribution to AMR than SCA veterinary respondents. Strict governance created by audit assurance programs overseeing food safety in Australia coupled with consumer drivers for minimising antibiotics and other drugs in the food chain has likely prompted a cultural shift in the use of antibiotics by Australian livestock veterinarians driven by market demands. Collaborations between the veterinary profession and the farming sector have been successful in countries such as the Netherlands, where partnerships overseen by Government focus on monitoring usage, application of reduction targets and increased focus on herd health and mandatory health plans. This has resulted in reductions of up to 58% in total antimicrobial consumption in food producing animals [24]. Australian Government agency reports [10] indicate that moderate progress has been made in reducing the use of high and medium rated antibiotics in agriculture and the total quantity of antibiotics used for animal health is very low compared to international standards. However, the intermittent publication of these reports, our current inability to closely monitor and report on antibiotic use at a sector or species level (as is done for example in the Netherlands) nor gauge the appropriateness of use such as in human medicine [37] makes it difficult to address public perceptions nationally or internationally, regarding the risk of AMR development in Australia via the food chain. This was highlighted in the current study and more broadly in a previous study by the authors [3] in which even other sectors of the Australian veterinary profession (SCA and equine) considered antibiotic use in the livestock sector to be a ‘moderate’ contributor to the issue of AMR.

Antibiotics were reported to be dispensed in a third of consultations by Australian veterinarians across all practice types. While it is recognized that the self-reporting of antibiotic use and its frequency is a limitation of this study, it does provide broad trends on antibiotic use. In SCA veterinarians, this is comparable to reports from the UK, in which antibiotics were prescribed in 21% of feline and 25% of canine consultations in an analysis of medical records across 374 practices [38] and a large study of approximately 1 million electronic medical records of cats and dogs in the UK (17.5–18.8%) [39]. In a unique analysis of pet insurance data in companion animals in Australia [40], the incidence rate of antimicrobial prescribing was 5.8 prescriptions per 10 dog years (95% CI 5.8–5.9 per 10 dog years) and 3.1 prescriptions per 10 cat years (95% CI 3.1–3.2 per 10 cat years), with antimicrobials of high importance accounting for 8% of all antimicrobials prescribed over the 4-year study. Further work interrogating the appropriateness of use as well as the frequency of prescribing, is required to inform future approaches to education within antimicrobial stewardship programs.

The frequent use of the potentiated aminopenicillin amoxicillin-clavulanate in SCA reported in our study is a consistent and concerning finding in surveys of small animal practitioners globally [39,41,42]. It has become the mainstay of empirical therapy for a wide range of clinical diseases in small animal practice [11,39] due, in part, to the lack of appropriate narrow-spectrum veterinary formulation alternatives and a normalisation of its widespread empirical use. In the current study, there was ‘moderate’ agreement by SCA veterinarians that ‘*narrow spectrum antibiotics were preferred over broad spectrum*’ but this was not reflected in frequencies of use, with three of the top five antibiotics used being broad spectrum and of medium to high importance to human and animal health [26]. Conversely, SCA veterinarians perceived their contribution to AMR as ‘minimal’ while considering antibiotic use in livestock and aquaculture industries to be a ‘moderate’ contributor. This disparity between knowledge and use may be driven by their reported patient-centric factors such as patient clinical signs and the critical nature of their illness as well as fear of missing an infection and difficulty in making an accurate diagnosis. The externalisation of the problem is also found across many professional sectors [3] and is a central consideration in changing the norm of antibiotic use. While further research is required to refine and better understand this disconnect between knowledge and use, the availability of appropriate narrow spectrum formulations of antibiotics for canine and feline patients is essential for the ongoing success of antimicrobial stewardship programs in small animal practice [43].

The use of horses as both companion and working animals but classified as a food producing animal by APVMA has restricted the registration of certain antibiotics of high importance for this species in Australia, such as the fluoroquinolones. Trimethoprim-sulphonamides are the most frequently administered antibiotics by equine veterinarians, consistent with previous studies [15,44] due to their use across a wide range of clinical diseases. While gentamicin and amikacin were grouped together in the survey, the routine use of gentamicin in horses for gram negative infections is the more likely reason for the median of ‘frequently’ as amikacin is rarely used, due to its high cost. Although reported as ‘rarely’, the use of rifampicin by equine clinicians (in conjunction with a macrolide antibiotic) for the treatment or prevention of ‘rattles’, a lower respiratory disease of foals caused by *Rhodococcus equi*, has been linked to the persistence of rifampicin-resistant ST612 methicillin-resistant *Staphylococcus aureus* in Australia and South Africa [45–47]. Equine veterinarians reported use of ceftiofur and fluoroquinolones (‘rare to sometimes’) despite the latter not being registered for horses in Australia. Equine veterinarians were more cognizant of their role in AMR, rating antibiotic use in horses as a ‘moderate’ problem however ongoing work in stewardship is required to improve the responsible use of antimicrobials in equine practice in Australia [15].

Surveillance of AMR in microbial isolates from clinically normal and sick humans and animals provide essential links to the potential impact of antibiotic usage [48]. Australia does not currently have a nationally funded AMR and antibiotic usage surveillance program focused on animals. A number of single time period surveys have been published which have confirmed a low public health risk in the food-animal sector related to resistance, including against critically important drugs such as fluoroquinolones [49,50]. Recent antimicrobial resistance surveillance studies in animals in Australia [47,50–52] have targeted common bacterial pathogens in human and animal medicine, namely *Staphylococcus* spp. and *Escherichia coli*, to determine the frequency of antimicrobial resistance in clinical isolates across Australian animal species. These have found higher frequencies of antibiotic resistance in clinical isolates from companion animals (including horses) than livestock species in these common bacterial species, with the frequency of fluoroquinolone resistance in companion animals comparable to human medicine, providing potential insights into the link between antimicrobial regulation, usage and resistance development. A national AMR surveillance network that reports on not only national veterinary clinical isolates, but the frequency of carriage of AMR within commensals and estimates the environmental impact of antibiotic use is required.

## Conclusion

This study confirms that across all veterinary practice types there is a need for affordable and rapid diagnostic testing, culture and AST to improve antimicrobial prescribing. Progress and compliance with antimicrobial stewardship principles is evident in livestock veterinarians whose use of critically important antibiotics was low and their awareness of their role in AMR was higher than SCA and equine veterinarians. The greatest disconnect between attitudes and practice was in SCA veterinarians. Access to national consensus antimicrobial guidelines, education on the importance rating of antimicrobial agents and the availability of narrow spectrum antibiotics of lower importance is essential for antimicrobial stewardship program success in all aspect of veterinary practice.

## Supporting information

S1 Survey Survey administered to Australian veterinarians regarding their attitudes to antibiotic use and antibiotic resistance. (DOCX)

S1 Table Data for the study of veterinarians’ attitudes to antibiotic use and antibiotic resistance. (XLSX)
