# Correction: Factors influencing the behaviour and perceptions of Australian veterinarians towards antibiotic use and antimicrobial resistance

**DOI:** 10.1371/journal.pone.0224844

**Published:** 2019-10-30

**Authors:** Jacqueline M. Norris, Annie Zhuo, Merran Govendir, Samantha J. Rowbotham, Maurizio Labbate, Chris Degeling, Gwendolyn L. Gilbert, Dale Dominey-Howes, Michael P. Ward

The affiliation for the first, eighth, and ninth authors is incorrect. Jacqueline M. Norris, Dale Dominey-Howes, and Michael P. Ward are not affiliated with #8 but with #7: Marie Bashir Institute for Infectious Diseases and Biosecurity, The University of Sydney, Sydney, New South Wales, Australia.
