# Supplementary material for: Factors influencing the behaviour and perceptions of Australian veterinarians towards antibiotic use and antimicrobial resistance
Source: PLoS One. 2019 Oct 10;14(10):e0223534. doi: 10.1371/journal.pone.0223534 (PMC6786536; doi:10.1371/journal.pone.0223534)
Supplement: S1 Survey — (DOCX) [file pone.0223534.s001.docx]

**Project: Attitudes towards antibiotic use and antibiotic resistance**

**Information about this study:**

**If you are currently registered to practise as a doctor, dentist OR veterinarian in Australia AND are able to prescribe antibiotics, you are invited to complete this survey. Your thoughts and participation are valuable to us.**

**What is this study about?**

We are undertaking a research study about prescribers’ views on antibiotic use, prescribing behaviour and antibiotic resistance in Australia. The survey is being conducted across multiple health and medical professions in order to gain a holistic understanding of attitudes towards antibiotic use and resistance. The findings from this study may be useful in assisting national policy-makers to revise and enhance national policy and education interventions about antibiotic use and antibiotic resistance in Australia.

**How much time will the study take?**

The questionnaire should take about 15-20 minutes to complete. The survey contains multiple choice and short answer questions about antibiotic prescribing and use, antibiotic resistance and sources of information about antibiotics.

**Do I have to be in the study? Can I withdraw from the study once I’ve started?**

Participation in this research study is voluntary. Completing the questionnaire will indicate your willingness to participate in this study. All of your responses will remain anonymous and treated in strict confidence. Your questionnaire responses cannot be withdrawn once they are submitted, as they are anonymous and therefore the researchers will not be able to tell which response is yours.

**Are there any benefits associated with being in the study?**

We cannot guarantee that you will receive any direct benefits from being in the study. However, your contribution will be valuable in generating new understanding to inform development of more effective policy and education in relation to antibiotic use, which will benefit the broader community.

At the end of the survey, you will have the opportunity to go into a prize draw to win an iPad.

**Will I be told the results of the study?**

The results of this questionnaire may be presented at future conferences, published in reports and publications and used in future studies. If you would like a general summary of the results of the study, please let us know by answering the relevant question at the end of the questionnaire. This feedback will be in the form of a one-page general summary. You will receive this summary after the study is finished.

At the end of the survey, there will be an option to indicate whether you would like to be contacted about future research surveys about antibiotics and antibiotic resistance at a later stage. We would value your participation in these studies. Participation in these later stages of the project is also completely voluntary.

**Thank you for taking the time to assist in this study.**

**What if I would like further information about the study?**

***If you have any questions or would like further information regarding the questionnaire or study in general, please contact:***

Dale Dominey-Howes (Chief Investigator)

Email: [dale.dominey-howes@sydney.edu.au](mailto:dale.dominey-howes@sydney.edu.au)

Telephone: +61 2 9351 6641


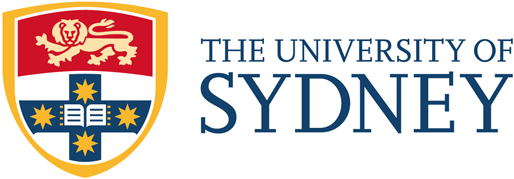


The study is being carried out by researchers at the University of Sydney and the University of Technology, Sydney led by Associate Professor Dale Dominey-Howes from the University of Sydney. This study is being funded by the Faculty of Science at the University of Sydney.

**What if I have a complaint or concerns about the study?**

This study has been approved by the Human Research Ethics Committee of the University of Sydney [2016/675]. If you have any concerns about the way this study is being conducted or you wish to speak to someone independent from the study, please contact the university using the details outlined below:

The Manager, Ethics Administration, The University of Sydney:

Telephone: +61 2 8627 8176

Email: [ro.humanethics@sydney.edu.au](mailto:ro.humanethics@sydney.edu.au)

# Section 1: Questions about you and your practice

1. **Are you currently registered to practise as any of the following in Australia?**

- Doctor
- Dentist
- Veterinarian
- None of the above – Please stop survey here. Unfortunately, the response you've just selected means that we cannot include you in this survey. Thank you for your interest and willingness to participate in the survey.

1. **In which state(s)/territories are you currently practising in Australia? (Tick all that apply)**

- ACT
- NSW
- NT
- SA
- QLD
- TAS
- VIC
- WA

1. **Which of the following best describes your current position? (Tick all that apply)**

- General veterinary practitioner
- Specialist veterinarian
- Government or industry employee
- University Faculty member
- Resident or Intern
- On plant veterinarian (OPV) at an abattoir
- Other (please specify):_____________________________________________

**Where do you currently spend your working time?**

| **Area** | **0-20% of my work time** | **21-40%** | **41-60%** | **61-80%** | **81-100% of my work time** |
| --- | --- | --- | --- | --- | --- |
| Private practice |  |  |  |  |  |
| University teaching hospital |  |  |  |  |  |
| University (research/teaching) |  |  |  |  |  |
| Diagnostic laboratory |  |  |  |  |  |
| Research laboratory |  |  |  |  |  |
| Not-for-profit veterinary practice (e.g. shelter) |  |  |  |  |  |
| Abattoir |  |  |  |  |  |
| Government |  |  |  |  |  |
| Industry |  |  |  |  |  |
| Other (please specify area and percentage of working time): |  |  |  |  |  |

1. **What is the postcode of your principal place of practice (where you predominantly practise)? _______________________________________**
2. **At which University/College did you complete your primary veterinary degree/qualification?**

- Charles Sturt University
- James Cook University
- Massey University New Zealand
- Murdoch University
- University of Adelaide
- University of Melbourne
- University of Queensland
- University of Sydney
- Other (please specify): __________________________________________

1. **In what year did you graduate from your primary veterinary degree?** (please specify) __________________________________________
2. **How many years in total post-graduation have you been working as a veterinarian (full-time or part-time)?** ___________________ years
3. **What is the highest level of education related to Veterinary Science you have completed? (Tick ONE only)**

- Undergraduate entry-level veterinary degree
- Graduate entry-level veterinary degree
- Graduate certificate or diploma
- Masters degree
- Specialist qualification or PhD
- Other (please specify):________________________________________

1. **Have you completed any specialty training OR are currently receiving specialty training?**

- No
- Yes – already completed specialty training
- Yes – currently receiving specialty training

**If YES, please specify the type of specialty training:** ________________________________________________________________

1. **In a typical working WEEK, approximately how many patients do you see? _________**
2. **In your work, have you ever supervised any of the following? (Tick all that apply)**

- Students
- Interns
- Residents
- New graduates
- None
- Other (please specify): __________________________________

1. **Do you speak a language(s) other than English with any of your clients?**

- No
- Yes - **please specify which language(s) AND the percentage (%) of your clients you speak it with:** ________________________________________

1. **What is your gender?**

- Female
- Male
- Other

1. **What is your age? _____________years**
2. **Thinking of your current or most recent veterinary workplace where you spend the most time working directly with animals, please estimate the proportion of time spent on each animal species (responses should total 100%).**

| **Animal species** | **Percentage %** |
| --- | --- |
| **If no animal handling ­ Please enter 100% here.** |  |
| **Dogs** |  |
| **Cats** |  |
| **Horses** |  |
| **Dairy cattle** |  |
| **Beef cattle** |  |
| **Sheep** |  |
| **Goats** |  |
| **Pigs** |  |
| **Poultry / other birds** |  |
| **Pocket pets (guinea pigs, ferrets, rabbits etc)** |  |
| **Fish** |  |
| **Australian wildlife** |  |
| **Zoo animals** |  |
| **Other (please specify species AND percentage of time):** |  |

**Definitions of key terms**

**For the purposes of this survey, the following terms have been defined as:**

***Antibiotics/Antibacterials****:* A subset of antimicrobial drugs that can be used to treat disease in humans and animals caused by bacteria.

***Antibiotic resistance****:* The ability of microbes/bacteria to resist lethal or inhibitory effects of antibiotics.

# Section 2: Questions about antibiotic prescribing and use

1. **In a typical working WEEK, how many patients do you administer/prescribe antibiotics to? _________**
2. **Which animal species do you treat the most? (please state ONE animal species) _____________**
3. **Thinking about the animal species you stated ABOVE, please indicate how often you currently prescribe/dispense/administer the following antibiotics to THAT species.**

|  | Never | Rarely | Sometimes | Frequently |
| --- | --- | --- | --- | --- |
| Penicillin G |  |  |  |  |
| Amoxicillin-clavulanate (e.g. Clavulox) |  |  |  |  |
| Amoxicillin/Ampicillin (e.g. Amoxyl) |  |  |  |  |
| Ticarcillin-clavulanate (e.g. Timentin) |  |  |  |  |
| Piperacillin-tazobactam |  |  |  |  |
| Flucloxacillin/Cloxacillin/Dicloxacillin |  |  |  |  |
| Cephalexin/Cephazolin (e.g. Keflex) |  |  |  |  |
| Ceftriaxone/Cefotaxime |  |  |  |  |
| Cefovecin (e.g. Convenia) |  |  |  |  |
| Ceftiofur (e.g. Excede, Naxcel) |  |  |  |  |
| Meropenem/Imipenem |  |  |  |  |
| Azithromycin |  |  |  |  |
| Clarithromycin |  |  |  |  |
| Erythromycin |  |  |  |  |
| Chloramphenicol |  |  |  |  |
| Ciprofloxacin |  |  |  |  |
| Enrofloxacin (e.g. Baytril) |  |  |  |  |
| Marbofloxacin (e.g. Zeniquin) |  |  |  |  |
| Pradofloxacin (e.g. Veroflox) |  |  |  |  |
| Clindamycin (e.g. Antirobe) |  |  |  |  |
| Doxycycline/Tetracycline (e.g. Vibravet) |  |  |  |  |
| Gentamicin/Amikacin |  |  |  |  |
| Metronidazole (e.g. Flagyl) |  |  |  |  |
| Rifampicin |  |  |  |  |
| Trimethoprim-sulfamethoxazole |  |  |  |  |
| Vancomycin |  |  |  |  |
| Linezolid |  |  |  |  |
| Polymyxin B |  |  |  |  |
| Other (please specify): __________________________ |  |  |  |  |

1. In your experience, to what extent do the following factors *influence* your decision **WHETHER or NOT TO** prescribe/administer antibiotics to an individual patient?

|  | No influence | Minimal influence | Moderate influence | Strong influence | N/A |
| --- | --- | --- | --- | --- | --- |
| Patient’s clinical signs/symptoms. |  |  |  |  |  |
| Patient’s medical and/or dental history. |  |  |  |  |  |
| Patient’s antibiotic use history. |  |  |  |  |  |
| If the patient is critically ill and/or immunocompromised. |  |  |  |  |  |
| Patient safety. |  |  |  |  |  |
| Guideline recommendations. |  |  |  |  |  |
| Culture and susceptibility test results. |  |  |  |  |  |
| My experience in managing similar problems. |  |  |  |  |  |
| Client expectations. |  |  |  |  |  |
| Peer/colleague/supervisor expectations. |  |  |  |  |  |
| Immediate patient relief. |  |  |  |  |  |
| Potential adverse/side effects of taking antibiotics. |  |  |  |  |  |
| Risk of promoting antibiotic resistance in bacteria in the patient. |  |  |  |  |  |
| Community-wide risks of antibiotic resistance. |  |  |  |  |  |

**Other factors** (please specify): __________________________________________________

1. In your experience, to what extent do the following factors influence your decision about **SELECTING WHICH** **ANTIBIOTIC** to prescribe/administer?

|  | No influence | Minimal influence | Moderate influence | Strong influence | N/A |
| --- | --- | --- | --- | --- | --- |
| Clinical signs/symptoms. |  |  |  |  |  |
| Patient’s antibiotic use history. |  |  |  |  |  |
| Cost of antibiotic. |  |  |  |  |  |
| Guideline recommendations. |  |  |  |  |  |
| Culture and susceptibility test results. |  |  |  |  |  |
| My previous clinical experience with antibiotic effectiveness. |  |  |  |  |  |
| Availability of antibiotic. |  |  |  |  |  |
| Client expectations/preferences. |  |  |  |  |  |
| Peer/colleague/supervisor expectations. |  |  |  |  |  |
| Spectrum of antibiotic activity (broad or narrow spectrum). |  |  |  |  |  |
| Route of administration. |  |  |  |  |  |
| Frequency of administration. |  |  |  |  |  |
| Size of tablet/capsule. |  |  |  |  |  |
| Duration of therapy. |  |  |  |  |  |
| Ease of administration. |  |  |  |  |  |
| Client compliance. |  |  |  |  |  |
| Potential adverse/side effects. |  |  |  |  |  |
| Potential to promote antibiotic resistance. |  |  |  |  |  |
| Manufacturer’s promotional material. |  |  |  |  |  |

**Other factors** (please specify): _________________________________________________

1. **Do you FEEL CONFIDENT doing the following actions? If you do not perform any of the following, please use N/A column.**

|  | Never | Rarely | Sometimes | Often | Always | N/A |
| --- | --- | --- | --- | --- | --- | --- |
| Making an accurate diagnosis of infection/sepsis. |  |  |  |  |  |  |
| Choosing the correct antibiotic. |  |  |  |  |  |  |
| Choosing the correct dose/dosage of antibiotic. |  |  |  |  |  |  |
| Using a combination of antibiotics if appropriate. |  |  |  |  |  |  |
| Choosing between routes of antibiotic administration (oral vs intravenous vs topical). |  |  |  |  |  |  |
| Interpreting microbiological/laboratory results. |  |  |  |  |  |  |
| Modifying/stopping antibiotic treatment according to microbiological investigations. |  |  |  |  |  |  |
| Planning the duration of the antibiotic treatment. |  |  |  |  |  |  |

1. **With respect to YOUR experience, how much of a BARRIER is each of the following to prescribing antibiotics appropriately? If not applicable to you, please use the N/A column.**

|  | Not a barrier | Somewhat a barrier | Moderate barrier | Significant barrier | N/A |
| --- | --- | --- | --- | --- | --- |
| Cost of culture and susceptibility tests. |  |  |  |  |  |
| Cost of some antibiotics. |  |  |  |  |  |
| Time pressure. |  |  |  |  |  |
| Lack of rapid diagnostic tests. |  |  |  |  |  |
| Difficulty of making an accurate diagnosis. |  |  |  |  |  |
| Lack of clear guidelines for some conditions. |  |  |  |  |  |
| Pressure from clients. |  |  |  |  |  |
| Pressure from colleagues/peers/supervisors. |  |  |  |  |  |
| Fear of missing an infection. |  |  |  |  |  |
| Fear of being blamed for not prescribing if antibiotics later prove necessary. |  |  |  |  |  |
| Fear of losing client to a different practice if I don’t prescribe. |  |  |  |  |  |
| Lack of client understanding about antibiotics. |  |  |  |  |  |
| Lack of my own understanding about antibiotics. |  |  |  |  |  |
| Language/cultural barrier when communicating with some clients. |  |  |  |  |  |
| Lack of time to search for information. |  |  |  |  |  |

**Other (please specify):**

___________________________________________________________________________

1. **What proportion of your clients do you have difficulty communicating with because of language/cultural barriers?**

- None
- Some
- Half
- Most

1. **Have you ever felt pressure/expectations from any of your clients to prescribe/administer antibiotics to their animals?**

- Yes
- No

1. **What tools/resources, if any, have you used to address pressure/expectations for antibiotics from clients? (tick all that apply)**

- Verbal explanations (e.g. of management plan)
- Posters in practice
- Brochures, pamphlets and information sheets for clients
- Materials translated in different languages
- Online resources
- None
- N/A
- Other (please specify): _______________________________________________________________

1. **Do you currently feel like you have enough skills/resources to deal with clients who pressure you to prescribe/administer antibiotics to their animals (when not indicated)?**

- Absolutely YES
- Mostly YES
- Neutral
- Mostly NO
- Absolutely NO

1. **Do you currently feel like you have enough skills/resources to discuss antibiotic resistance with clients?**

- Absolutely YES
- Mostly YES
- Neutral
- Mostly NO
- Absolutely NO

1. **What resources/tools do you think would better help you in dealing with clients who expect/pressure you to prescribe/administer antibiotics to their animals in the future?**

__________________________________________________________________________________________________________________________________________

1. **Are there any antibiotics that you don’t feel comfortable prescribing/using because of their higher risk of promoting antibiotic resistance?**

- No
- Yes **- please specify which antibiotic(s):** _________________________________________________ _________________________________________________

_________________________________________________

# **Section 3: Questions about information sources**

1. **How *useful* are the following information sources to YOU on appropriate antibiotic use? If the sources are currently *not available* or *not familiar* to you, please use the last column.**

|  | Not useful | Somewhat useful | Useful | Very useful | Not available/ Not Familiar |
| --- | --- | --- | --- | --- | --- |
| Scientific/clinical journals |  |  |  |  |  |
| MIMS |  |  |  |  |  |
| Textbooks or Medicines Handbooks |  |  |  |  |  |
| Applications on smart phone or tablet |  |  |  |  |  |
| National antibiotic use/prescribing guidelines |  |  |  |  |  |
| Online search engines (e.g. Google, Bing) |  |  |  |  |  |
| Policy/guidelines of practice/hospital |  |  |  |  |  |
| My clinical experience |  |  |  |  |  |
| My entry-level university degree/education |  |  |  |  |  |
| My postgraduate education/training |  |  |  |  |  |
| My specialty education/training |  |  |  |  |  |
| Continuing Education/Training |  |  |  |  |  |
| Antibiotic labels |  |  |  |  |  |
| Colleagues/peers/supervisors |  |  |  |  |  |
| Advice from a microbiologist |  |  |  |  |  |
| Advice from an Infectious Diseases specialist |  |  |  |  |  |
| Advice from a Pharmacist |  |  |  |  |  |
| Advice from an Infection Control specialist |  |  |  |  |  |
| Antimicrobial stewardship team |  |  |  |  |  |
| Pharmaceutical company representatives |  |  |  |  |  |
| Information from professional associations |  |  |  |  |  |
| Government reports |  |  |  |  |  |
| Media reports |  |  |  |  |  |

**Other useful information sources you commonly use (please specify): ___________________________________________________________________________**

**___________________________________________________________________________**

1. **Do you think you have enough information sources about antibiotics when you need it?**

- Yes
- No – Please specify what other sources you think would be useful to you: ______________________________________________________________________________________________________________________________________________________________________________________________________

1. **Does your principal place of practice have a practice policy on antibiotic prescribing?**

- Yes
- No
- Unsure

1. **Have you had any Continuing Education/Professional Development in the past 3 years where the use of antibiotics OR antibiotic resistance was a major focus?**

- Yes
- No

**If YES, how was the training delivered? (Tick all that apply)**

- Lecture(s)
- Workshop
- Conference
- Informal education in the clinical workplace
- One-on-one consultation
- Online/web-based learning
- Application on smart phone or tablet
- Self-directed learning
- Other (please specify):_____________________________

1. **In what form(s) would you most prefer future continuing education to be delivered? (Please tick up to 3 options)**

- Lecture(s)
- Workshop
- Conference
- Informal education in the clinical workplace
- One-on-one consultation
- Online/web-based learning
- Self-directed learning
- Application on smart phone or tablet
- Other (please specify): __________________________________

1. **Are you a member of or affiliated with any professional organisations, associations or colleges?**

- No
- Yes

**If YES, please specify:**

___________________________________________________________________________

1. **How helpful do you think the following measures are/would be in supporting appropriate antibiotic prescribing in YOUR place of practice?**

|  | Not helpful | Somewhat helpful | Helpful | Very helpful | Unsure |
| --- | --- | --- | --- | --- | --- |
| More education/training on antibiotic prescribing. |  |  |  |  |  |
| Better public awareness about antibiotic resistance. |  |  |  |  |  |
| Access to rapid diagnostic tests. |  |  |  |  |  |
| Computer/electronic device aided prescribing. |  |  |  |  |  |
| Access to timely information on antibiotic resistance and susceptibility patterns. |  |  |  |  |  |
| Regular audit and personal feedback on my prescribing. |  |  |  |  |  |
| Antimicrobial stewardship programs. |  |  |  |  |  |
| Restriction on prescription of *certain* antibiotics (requiring specialist opinion/by governing authority). |  |  |  |  |  |
| Restriction on prescription of *all* antibiotics (requiring specialist opinion/by governing authority). |  |  |  |  |  |

**Other measures you think would be useful:**

__________________________________________________________________________________

__________________________________________________________________________________

# **Section 4: Questions about antibiotics and antibiotic resistance**

1. **For each of the following statements, please indicate to what extent you agree/disagree with the statement.**

|  | Strongly disagree | Disagree | Neutral | Agree | Strongly agree |
| --- | --- | --- | --- | --- | --- |
| Antibiotic use in one patient may weaken its effectiveness in the same individual in the future. |  |  |  |  |  |
| Antibiotic use in one patient may weaken its effectiveness for other patients in the future. |  |  |  |  |  |
| New antibiotics will be developed that will keep up with the problem of antibiotic resistance. |  |  |  |  |  |
| A single course of antibiotics can cause antibiotic resistance. |  |  |  |  |  |
| Antibiotic-resistant bacteria may last a year in a patient after single use of an antibiotic. |  |  |  |  |  |
| In recent years I have become more aware of the impacts of antibiotic resistance. |  |  |  |  |  |
| The antibiotics I prescribe contribute to the problem of antibiotic resistance. |  |  |  |  |  |
| Antibiotic resistance will become a greater clinical problem in the future than it is today. |  |  |  |  |  |

1. **Have you ever seen antibiotic-resistant infections in patients?**

- Yes
- No
- Unsure

1. **Have you ever experienced treatment failure with antibiotics?**

- Yes
- No
- Unsure

1. **To what extent do you think the following factors contribute to the issue of antibiotic resistance?**

|  | No contribution | Minimal | Moderate | Significant contribution | Unsure |
| --- | --- | --- | --- | --- | --- |
| Too many antibiotic prescriptions. |  |  |  |  |  |
| Too long durations of antibiotic treatment. |  |  |  |  |  |
| Too low doses of antibiotics. |  |  |  |  |  |
| Antibiotic use in minor or self-limited illnesses. |  |  |  |  |  |
| Not removing the site/source of infection (e.g. medical devices, catheters, tooth). |  |  |  |  |  |
| Prescribing an antibiotic when benefit to patient is uncertain. |  |  |  |  |  |
| Continuing empirical antibiotic therapy without appropriate laboratory investigation. |  |  |  |  |  |
| Prescribing broad spectrum antibiotics when equally effective narrower spectrum antibiotics are available. |  |  |  |  |  |
| Patients/clients not finishing prescribed course of antibiotic. |  |  |  |  |  |
| Patients/clients using antibiotics from previously unfinished prescriptions. |  |  |  |  |  |
| Poor hand hygiene. |  |  |  |  |  |
| Poor environmental hygiene in healthcare settings. |  |  |  |  |  |
| Environmental contamination with antibiotic waste (e.g. from farms, hospitals, homes). |  |  |  |  |  |
| Transfer of resistant bacteria between humans, animals and environment. |  |  |  |  |  |

1. To what extent do you think **CURRENT LEVELS OF ANTIBIOTIC USE** in the following contribute to the issue of antibiotic resistance**?**

|  | No contribution | Minimal | Moderate | Significant contribution | Unsure |
| --- | --- | --- | --- | --- | --- |
| In my principal place of practice. |  |  |  |  |  |
| In general medical practice. |  |  |  |  |  |
| In human dental practice. |  |  |  |  |  |
| In human hospitals. |  |  |  |  |  |
| In nursing homes/aged care facilities. |  |  |  |  |  |
| In livestock. |  |  |  |  |  |
| In companion animals (i.e. pets, horses). |  |  |  |  |  |
| In aquaculture (e.g. fish farms, crustaceans) |  |  |  |  |  |
| Unregulated use of antibiotics globally. |  |  |  |  |  |

1. **How much of a problem do you think antibiotic resistance is to the HEALTH of the following in Australia?**

|  | Not a problem | Minor problem | Moderate problem | | Significant problem | Unsure |
| --- | --- | --- | --- | --- | --- | --- |
| Myself |  |  |  |  | |  |
| My patients |  |  |  |  | |  |
| Patients in human dental hospitals |  |  |  |  | |  |
| Patients in human medical hospitals |  |  |  |  | |  |
| The general public |  |  |  |  | |  |
| Residents in nursing homes/aged care facilities |  |  |  |  | |  |
| Patients in veterinary clinics |  |  |  |  | |  |
| Horses |  |  |  |  | |  |
| Dogs and cats |  |  |  |  | |  |
| Livestock and food animal industry |  |  |  |  | |  |

**Other (please specify): __________________________________________________**

1. **How much of a problem do you think antibiotic resistance is to the HEALTH of the following?**

|  | Not a problem | Minor problem | Moderate problem | | Significant problem | Unsure |
| --- | --- | --- | --- | --- | --- | --- |
| People in developing countries |  |  |  |  | |  |
| People in developed countries |  |  |  |  | |  |

1. **In your opinion, how important are the roles of each of the following in managing/preventing the issue of antibiotic resistance?**

|  | Not important | Somewhat important | Moderately Important | Very important | Extremely important |
| --- | --- | --- | --- | --- | --- |
| Myself |  |  |  |  |  |
| My immediate colleagues/peers |  |  |  |  |  |
| My clients/patients |  |  |  |  |  |
| The general public |  |  |  |  |  |
| Pet owners |  |  |  |  |  |
| Farmers and food producers |  |  |  |  |  |
| Veterinarians |  |  |  |  |  |
| Dentists |  |  |  |  |  |
| General medical practitioners (GPs) |  |  |  |  |  |
| Hospital doctors |  |  |  |  |  |
| Nurses |  |  |  |  |  |
| Pharmacists |  |  |  |  |  |
| Scientists |  |  |  |  |  |
| Governments |  |  |  |  |  |
| Pharmaceutical companies |  |  |  |  |  |
| The Media |  |  |  |  |  |
| Global organisations (e.g. World Health Organisation, World Organisation for Animal Health) |  |  |  |  |  |

**Others (please specify):** ______________________________________________________

**Is there anything else you would like to tell us about? Any feedback?**

_________________________________________________________________________________________________________________________________________________________________________________________________________________________________

**Thank you for taking the time to complete the questionnaire. Your participation is appreciated and highly important to this study.**

If you would like a general summary of the findings of this study at the conclusion of the research, please provide your contact details below. Our team of researchers would like to further this research on prescriber attitudes on antibiotics and antibiotic resistance in the future. Please also indicate below whether you give permission to researchers to be contacted regarding future engagement in research (e.g. in future surveys). Please note that participation in this future research will also be completely voluntary. This information will be kept in strict confidence by the researchers. Please indicate below whether you would like to enter the prize draw for the chance to win an iPad.

**The following questions are OPTIONAL. Any information you provide here will not be linked to your responses to the questionnaire. All information you provide here will be kept strictly confidential.**

1. **I would like to receive a general summary of the findings at the conclusion of the research.**
   - - - Yes - Please provide your contact information below
       - No
2. **I give permission for the researchers to contact me in the future in relation to future surveys and research.**
   - - - Yes – Please provide your contact information below
       - No
3. **Contact information:**

Name: __________________________________________

Email address: ________________________________________________________

Best contact number: ___________________________________________________

1. **Would you like to enter the prize draw for the chance to win an iPad? The winner will be drawn from a random draw of entries received, at the close of the survey.**
   - - - Yes – Please enter your contact information in the comment box below (if different to your contact details above)
       - No

**Please enter your contact details here for entry into the prize draw (name, email address, phone number):**

__________________________________________________________________________________________________________________________________________

_________________________________________________________________________________________________________________________________________________________________________________________________________________________________

**Thank you for your time and participation.**
